# The Sensory Components of High-Capacity Iconic Memory and Visual Working Memory

**DOI:** 10.3389/fpsyg.2012.00355

**Published:** 2012-09-25

**Authors:** Claire Bradley, Joel Pearson

**Affiliations:** ^1^The School of Psychology, The University of New South WalesSydney, NSW, Australia; ^2^Ecole Normale Supérieure de CachanCachan, France

**Keywords:** iconic memory, visual working memory, early visual memory, low-level visual features

## Abstract

Early visual memory can be split into two primary components: a high-capacity, short-lived iconic memory followed by a limited-capacity visual working memory that can last many seconds. Whereas a large number of studies have investigated visual working memory for low-level sensory features, much research on iconic memory has used more “high-level” alphanumeric stimuli such as letters or numbers. These two forms of memory are typically examined separately, despite an intrinsic overlap in their characteristics. Here, we used a purely sensory paradigm to examine visual short-term memory for 10 homogeneous items of three different visual features (color, orientation and motion) across a range of durations from 0 to 6 s. We found that the amount of information stored in iconic memory is smaller for motion than for color or orientation. Performance declined exponentially with longer storage durations and reached chance levels after ∼2 s. Further experiments showed that performance for the 10 items at 1 s was contingent on unperturbed attentional resources. In addition, for orientation stimuli, performance was contingent on the location of stimuli in the visual field, especially for short cue delays. Overall, our results suggest a smooth transition between an automatic, high-capacity, feature-specific sensory-iconic memory, and an effortful “lower-capacity” visual working memory.

## Introduction

Short-term sensory memory has been proposed to be the first step in forming more high-level and permanent memory stores that support behavior. For example, was the arrow on a road-sign pointing left or right? Was it red or green? Choosing the right answer to such questions could have a significant behavioral impact. This early visual memory can be split into two primary components: a high-capacity, short-lived iconic memory (Sperling, 1960; Coltheart, 1980) followed by a limited-capacity visual working memory that can last many seconds (Pasternak and Greenlee, 2005; Fukuda et al., 2010; Keogh and Pearson, 2011).

Although these two types of memory may, by definition, overlap in their temporal decay profiles, they are often studied separately, and seem to have different properties. Iconic memory is thought to last only a few 100 ms after perception and is often considered automatic (Neisser, 1967; Coltheart, 1980). Visual working memory, on the other hand, is considered a more effortful process that tends to last many seconds and requires active maintenance of information (Pasternak and Greenlee, 2005; Awh et al., 2006; Fougnie, 2008). While iconic memory is thought to have a virtually unlimited capacity, visual working memory is thought to have a severely limited-capacity that differs across individuals (see Brady et al., 2011 for a review; Vogel and Machizawa, 2004; Fukuda et al., 2010). Here, we examined visual short-term memory across a range of storage durations from 0 to 6 s that enabled us to study both iconic and visual working memory with one set of low-level visual stimuli.

Single items of color, form and motion seem to be retained in visual working memory with great precision and show little decay over periods of up to 30 s (Nilsson and Nelson, 1981; Vogels and Orban, 1986; Magnussen and Greenlee, 1992; Blake et al., 1997; Magnussen et al., 1998; Pasternak and Greenlee, 2005). These low-level visual features seem to be remembered with high precision, sometimes close to the resolution of perception when only a single item is held in memory (Blake et al., 1997; Magnussen and Greenlee, 1999; Magnussen, 2000; Fukuda et al., 2010). The stimulus features that affect perceptual detection and discrimination tend to have little influence on the decay functions of the related memory for a single item (Blake et al., 1997; Magnussen and Greenlee, 1999).

Little is known, however, about the perceptual properties of iconic memory. Numerous studies suggest that the information stored in iconic memory displays low-level characteristics. Specifically, it seems to be degraded by visual masks (Averbach and Coriell, 1961; Spencer, 1969; Turvey, 1973; Phillips, 1974; Gegenfurtner and Sperling, 1993) and may be dependent on spatial location (Phillips, 1974; McRae et al., 1987). However, most research on iconic memory utilized “high-level” alphanumeric stimuli such as letters or numbers, which often carry semantic content. For example even when color or luminance was of primary interest for investigation, alphanumeric figures were still used as stimuli (Coltheart et al., 1974).

To the best of our knowledge no study has yet examined the visual features of color, form, and motion within the same subjects using stimuli without higher-level semantic content. Here, we sought to investigate iconic memory and visual working memory for the low-level visual features of color, orientation, and motion, without any potential influence from high-level semantic information in the stimuli.

We report that color, orientation, and motion information can be stored in early visual memory. However, less motion information seems to be retained in iconic memory compared to color or orientation. Additionally, we found partial memory retention of 10 items at 1 s that relies on active attentional resources. Such active encoding and maintenance suggest the involvement of visual working memory for periods of 1 s or more. Likewise we showed a decay of retinotopic orientation information with longer retention intervals, suggesting ascension in the visual processing hierarchy with longer storage times. Together these results suggest a smooth transition from iconic to visual working memory mechanisms across retention times of 1 s.

## Materials and Methods

### General methods

#### Participants

Five subjects took part in this study (three females, two males, age 21–28, mean 23), four of which were naive to the purpose of the study. All had normal or corrected-to-normal vision and gave informed written consent before taking part in the experiment. The participants had normal color vision, as assessed by pseudo-isochromatic plates for testing color perception (Richmond Products). Four participants took part in all experiments, while one took part in only one experiment (Experiment 1, *N* = 4; Experiment 2, *N* = 4, Experiment 3, *N* = 5).

#### Apparatus

The experiment was performed using the Psychophysics Toolbox (Brainard, 1997) for MATLAB running on a MacPro computer. Stimuli were displayed on a calibrated 20′ SONY Multiscan G520 CRT with a monitor resolution of 1024 × 768 and a refresh rate of 100 Hz. Responses were recorded using the right and left arrows of a keyboard. Participants sat in a dimly lit room, 57 cm from the screen, with their head on a head- and chin-rest allowing their eyes to be aligned with the center of the screen.

#### Flicker photometry

Participants were presented with two alternating chromatic fields (3.6° diameter disks): a reference field and a variable field (Wagner and Boynton, 1972; Jiang et al., 2007). The two fields alternated at 25 Hz, creating a fused percept when the colors were equiluminant and a flickering percept when they were not. Participants were asked to increase and decrease the luminance of the variable field, until the flicker disappeared. This procedure was repeated three times and yielded the value of luminance required for the two colors to be perceptually equiluminant. Six color points across the red-green RGB color space were chosen and tested in pairs, each of them was in turn variable and reference. These six reference points allowed us to extrapolate equiluminance parameters by means of a linear relationship for all the colors used in this study.

#### Stimuli

**Figure 1:**
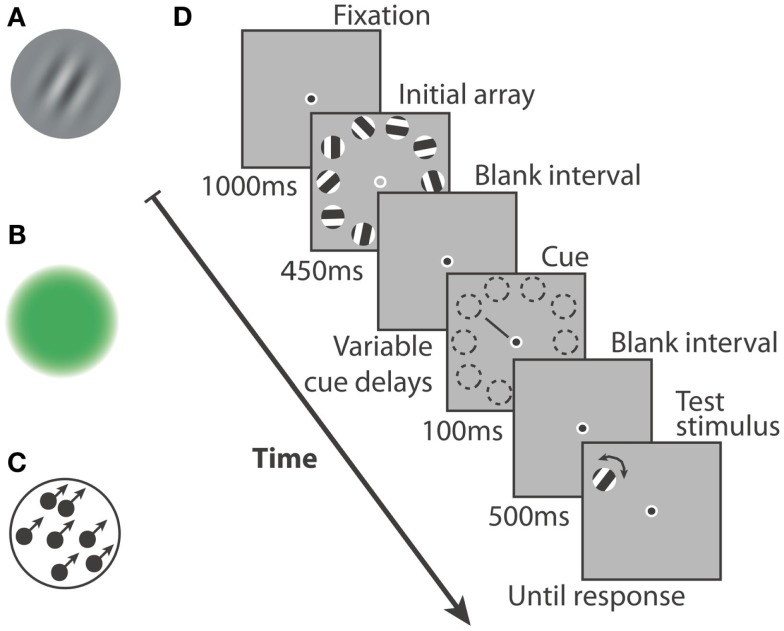
**Three types of stimuli were used in this study: (A)** Gabor patches that could vary in orientation, **(B)** uniform hue patches that varied smoothly from red to green, **(C)** dot motion kinetograms, moving with 100% coherence, that could vary in direction of motion. **(D)** A single trial had the following structure: after maintaining fixation, participants were shown an array of 10 stimuli for 250 ms (color or motion) or for 450 ms (orientation). From the offset of the initial array and with variable delay, a cue could appear. It would point at one of the 10 stimulus locations for 100 ms. After an interval of 500 ms, a test stimulus would appear at the cued location and the participant had to signal the direction of change in feature space (two alternative forced choice).

Stimuli consisted of 10 circular patches that could all be color, orientation, or motion stimuli (Figures 1A–C). Regardless of their type, the patches had a diameter of ∼2.5° and were evenly spaced on a 15.6° diameter circle centered on a fixation point (bulls eye fixation point: 0.3° inner diameter, 0.5° outer) positioned in the center of the screen. The edges of the color and orientation patches were smoothed with a Gaussian mask. They were displayed against a gray background. A thin black line (length: 1.8°) was used as a cue to point toward one of the 10 locations. The size and positions of items on the screen were invariant across all experiments. A test stimulus was used, that had the same location and properties as one of the 10 original stimuli apart from a modification in the relevant feature that was tested. For each feature, the value of this modification was adjusted in a pilot experiment so that performance with no memory delay was approximately 90%. This was to ensure that the difficulty of the task in the initial condition was equal between features.

Orientation stimuli (Figure 1A) were Gabors with a 50% Michelson contrast, a spatial frequency of 1.8 cycles per degree and an orientation picked out from a set of 10 possible values (180° divided by 10). The phase of the gratings was shifted by 180° at a rate of 25 Hz, resulting in flickering but perfectly visible gratings. This procedure reduced the formation of strong after-images, which rendered the task overly easy. Indeed, afterimages and visual persistence were not the investigative aim of the current study. The modification applied to the test stimulus was a change in orientation of ±12°.

Color stimuli (Figure 1B) consisted of colored disks, each defined by specific values of the red and green channels in RGB coordinates and a null value of the blue channel. Perceptual equiluminance was measured for each participant using flicker photometry. The stimuli and background colors were adjusted accordingly. A Gaussian mask was fit to the disks, thus creating the color patch stimuli. The color of one of them was shifted by 8.6% of the full color space toward green or red in order to create the test stimulus. Test stimuli which had a color obviously close to an extreme caused a positive bias because that color could only result from an increase in green (pure green stimulus) or in red (pure red stimulus). They were therefore included in the experiment but removed from the analysis (margin: maximum and minimum values minus 8.6% of the color space).

Dot motion displays were used as motion stimuli (Figure 1C). In each patch, 40 black dots (3 × 3 pixels) moved at a speed of 11°/s against a gray background. Motion coherence was 100%, that is, within one patch, all dots moved along imaginary lines of exactly the same orientation. In order to conserve dot density, dots were re-drawn on the opposite-side upon exiting the invisible boundaries of the patch. The direction of motion was randomly chosen from a set of 10 values (360°/10). One of them was modified by ±12° to obtain the orientation of the test stimulus.

#### Procedure

Each trial began with a 1000 ms blank screen with a fixation point in the center (Figure 1D). Ten stimuli then appeared on the screen for 250 ms (color or motion stimuli) or for 450 ms (orientation stimuli). The initial array presentation was followed by a blank retention interval (fixation point alone) until the cue pointed to one of the 10 stimulus locations (100 ms). The time between initial array offset and cue onset was randomly chosen from a list of cue delays. After another blank interval of 500 ms, the test stimulus appeared at the cued location. In the case of color stimuli, subjects were required to answer the following question: “Is the test stimulus more red or more green than the initial cued stimulus?” by pressing the right arrow for the first answer, or the left arrow for the second one. For the orientation and motion stimuli, the question was: “Is the test stimulus rotated clockwise or anti-clockwise compared to the initial stimulus?” and was answered by a right arrow or a left arrow button-press, respectively. The test stimulus stayed on-screen until the participant gave his answer, and the following trial then began. It is important to note that presentation time (either 250 or 450 ms in this experiment) does not seem to influence performance (see results). In fact, iconic memory is thought to be independent of the duration of stimulus presentation (Sperling, 1960; Dick, 1974), at least within our time range of 200–400 ms (Di Lollo and Dixon, 1992).

### Experiment 1

#### Procedure

Trials had the structure described above. Each feature (color, orientation, and motion) was tested separately, in a block design. Cue delays were chosen from the following list: (0, 100, 200, 300, 400, 700, 1000, 2000, 3000, 4000, 6000 ms). An additional perceptual condition was tested, in which the cue was displayed at the onset of the initial stimulus array, during initial array presentation. In order to minimize subject fatigue the range cue delays were performed in two blocks, in an interleaved manner (short and long delays mixed within a run). A typical run would thus have 120 trials in it (6 delays × 10 locations × 2 possible questions) and would last approximately 8 min. Five runs were collected for each delay, which corresponds to one hundred trials per delay. Data acquisition from each subject was distributed in time over a few days to a few weeks in pseudo random order.

### Experiment 2

#### Stimuli

The same color, orientation, and motion stimuli as before were used in this experiment. In addition to these stimuli, a distractor task was used to manipulate attention and mental resources. Subjects had to monitor a rapid serial visual letter stream at fixation, made of black letters (Helvetica font, normal style, text size: 0.4° in width). The letters were displayed in lower case and all letters of the alphabet could occur.

#### Procedure

Only one 1000 ms cue delay was used in this experiment. The procedure was the same as before, except that during the retention interval, two situations could arise (Figure 4A). On half of the trials, the interval was blank, with only a fixation point at the center of the screen. On the other half (distractor task condition), after 100 ms of blank screen, letters were presented in rapid succession (120 ms per letter, no separation) for 900 ms. The target letters, “C” and “V,” did not appear in the two first letters and had then to be separated from the next occurrence of a target letter by either 2, 4, or 6 letters. Subjects were instructed to press the C or V keys on the keyboard as soon as they detected the corresponding letter; their answer was recorded if it fell in a 425 ms time-window after letter presentation. The difficulty of this task required subjects to attend to the center of the screen. A tone was sounded before the beginning of each trial; a low-pitched tone indicated a normal trial, a high-pitched tone denoted a trial with a distractor task. The two conditions, with or without distractor task were interleaved in a run of 40 trials (10 locations × 2 possible questions × 2 letter conditions) that lasted 3 min. A total of 8 runs, equivalent to 160 trials, were collected for each condition. Color, orientation, and motion were again tested in separate blocks.

### Experiment 3

#### Stimuli

Only orientation stimuli previously described were used in this experiment.

#### Procedure

The procedure was the same as in Experiment 1, except that three different locations were possible for the test stimulus: either the same location (location at which the line pointed), or central (at fixation), or a location directly opposite the cued location e.g., the position on the peripheral ring of stimuli directly opposite. The test stimulus (at all locations) was always to be judged compared to the one in the original array at the cued location. The test stimulus remained on the screen until the subject responded. Each block consisted of 180 trials (3 delays × 10 positions × 2 possible questions × 3 test locations) and lasted approximately 10 min; 8 runs were used, which corresponds to 160 trials per condition.

## Results

In order to test early visual memory for low-level visual features, we measured performance on a memory task over the range of 0–6 s after stimulus presentation. Participants viewed an array of 10 stimuli. After the array disappeared, one of the 10 locations was cued and a test stimulus appeared. Participants had to compare the two stimuli (cued and test) and signal the direction of change in feature space (2AFC). Three stimulus features were tested separately: color, orientation, and motion.

**Figure 2:**
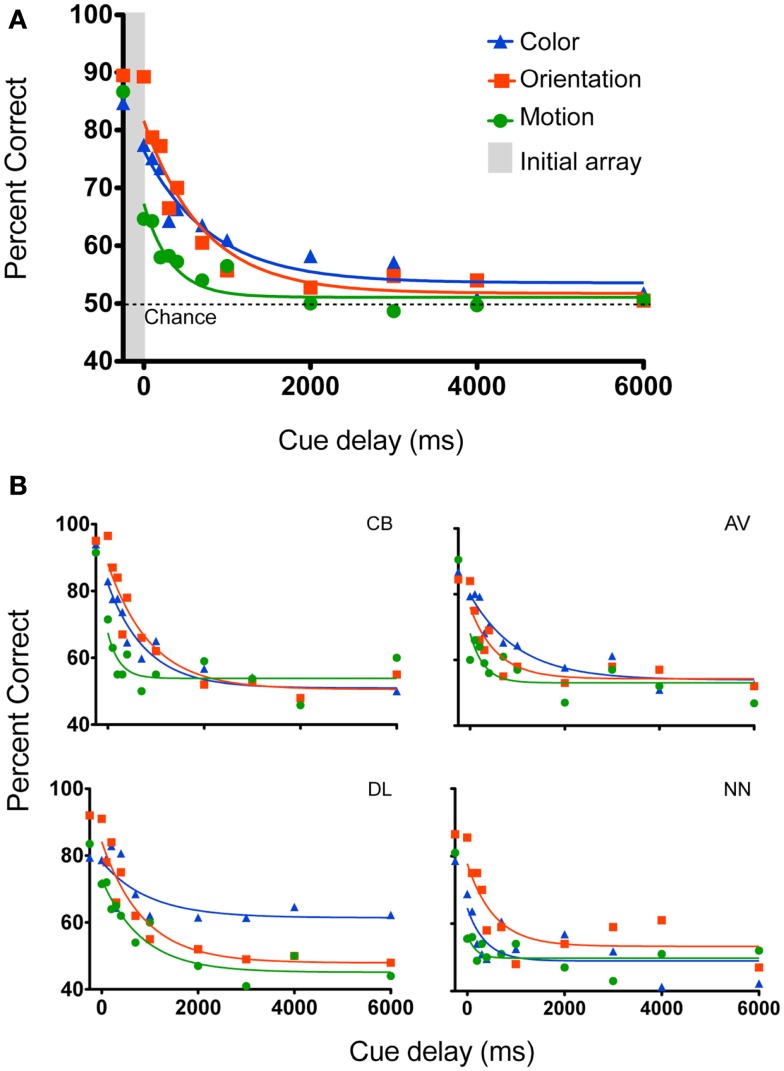
**Experiment 1 tested memory for color, form, and motion for the following cue delays after stimulus offset: (simultaneous, 0, 100, 200, 300, 400, 700, 1000, 2000, 3000, 4000, 6000 ms)**. **(A)** Mean data. The points within the gray vertical bar show performance when the cue was presented during the initial array. An exponential-decay function was fit to the data. Performance declined over time and was worse for motion than for color or orientation. **(B)** Individual subject data, curves represent best-fitting one-phase exponential-decay functions.

Figure 2 shows performance as a function of cue delay. When the cue was presented at the same time as the initial array (data points in the gray vertical bar), performance was high (85–90%) for all three conditions, with no significant difference between conditions [*F*(2, 6) = 1.5, *P* = 0.29]. This suggests that the task of identifying the change in feature space was relatively easy when performed without the need to hold the array in memory. It also suggests that the task difficulty was initially equivalent across all three features. Hence, any change in performance with memory storage should be due to mechanisms of “memory” and not perception.

**Figure 3:**
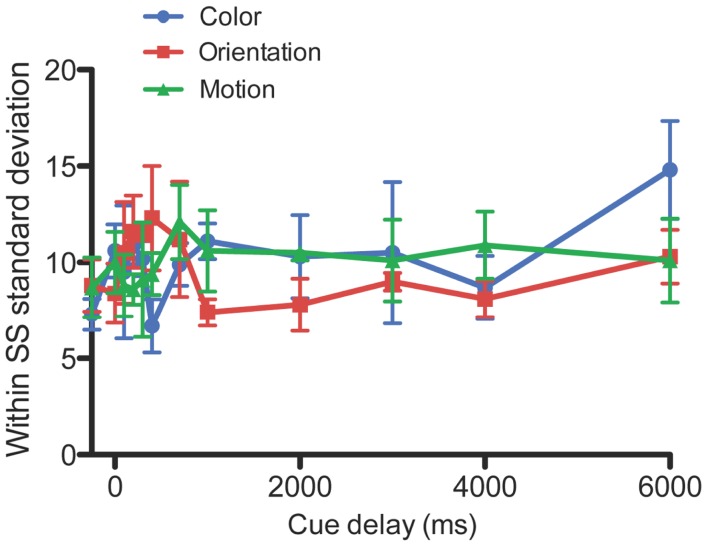
**Within subject standard deviation for color, orientation, and motion**. The standard deviation across the five blocks for each individual subject, averaged across the four subjects. Error bars denote standard errors of the mean.

To characterize the temporal dynamics of memory, a one-phase exponential-decay function was fit to the data using best fit least squares method (Lu et al., 2005; Graziano and Sigman, 2008; Kuhbandner et al., 2011; Figure 2). The goodness of fit between the observed and predicted values was reasonable (Color: *R* = 0.79; Orientation: *R* = 0.91; Motion: *R* = 0.87). When subjects were instructed to remember all 10 items, performance declined over time [main effect of cue delay *F*(11, 33) = 41.6, *P* < 0.001]. From the stimulus offset onward, accuracy was lower for the motion stimuli [main effect of feature *F*(2, 6) = 8.45, *P* = 0.018]. Performance in the color and orientation conditions was statistically the same [*F*(1, 3) = 0.188, *P* = 0.69], even though the initial performance *Y0* (Table 1) was lower for color than for orientation. As the differences in performance are mainly for shorter delays (0–1000 ms), these data suggest that iconic memory might hold less motion information than color or orientation. At cue delays of more that 2 s performance approached chance levels (∼50% correct) for all three features, suggesting that participants did not have enough information left to support performance. Figure 2B shows individual data plots for the participants, while Figure 3 shows the within subject standard deviation across the 5 blocks of trials. Accuracy for motion discrimination was lower than for color or orientation consistently across all participants. To the best of our knowledge this is the first study to examine the visual features of color, form and motion within the same subjects using stimuli without higher-level semantic content.

**Table 1:** **Exponential fit parameters for mean data, expressed as the best fit value and its 95% confidence interval (95% CI)**.

	Y0	Plateau	Tau	Span
Color	76.5 ± 3.45	53.6 ± 4.53	808.6 ± 600.0	22.87 ± 5.87
Orientation	81.6 ± 2.73	51.8 ± 3.42	715.8 ± 236.2	29.82 ± 4.57
Motion	67.3 ± 2.96	51.1 ± 2.58	327.5 ± 115.9	16.19 ± 2.93

*A one-phase decay equation of the form *Y* = (*Y0 − Plateau*) × *exp(−t/Tau)* + *Plateau* was fitted to the data; where *Y* is performance (% correct responses), *Y0* is performance at memory onset; *Plateau* is the performance reached for long delays, *t* is time after stimulus offset (ms) and *Tau* is the time constant (ms). The span corresponds to (Y0 – Plateau)*.

We ran a control experiment to investigate any effect of display time using the orientation stimuli. For 4 different display time durations (80, 200, 500, 800 ms) there was no main effect of display time [within-subjects ANOVA *F*(3, 6) = 0.45, *P* = 0.73], although there was still a main effect of cue delay [*F*(6, 6) = 13.47, *P* = 0.004; data not shown]. Hence, our data fit well with previous work demonstrating that display time does not significantly alter iconic memory (Sperling, 1960; Dick, 1974).

Memory at retention durations of 1 or 2 s is often considered to be visual working memory. However, visual working memory is thought to have a capacity limit well below 10 items (see Brady et al., 2011 for a review). In fact, research suggests that visual working memory may have an upper capacity limit of between three and five items (Luck and Vogel, 1997; Cowan, 2001; Vogel et al., 2001). If visual working memory is responsible for performance at 1 s, it might hold some information about the 10 stimuli and might therefore display a higher capacity than traditionally assumed. It might also be the case that performance at 1 s is supported by a long-lasting iconic memory. In order to characterize the memory process supporting performance 1 s after stimulus offset, we used a property that typically distinguishes iconic memory and visual working memory: attentional resources. Iconic memory has been described as an automatic process (Neisser, 1967), while visual working memory seems to involve attentional resources (Awh et al., 2006; Fougnie, 2008). Accordingly, introducing an attentionally demanding task during the retention interval might not disrupt iconic memory storage, but may affect visual working memory.

**Figure 4:**
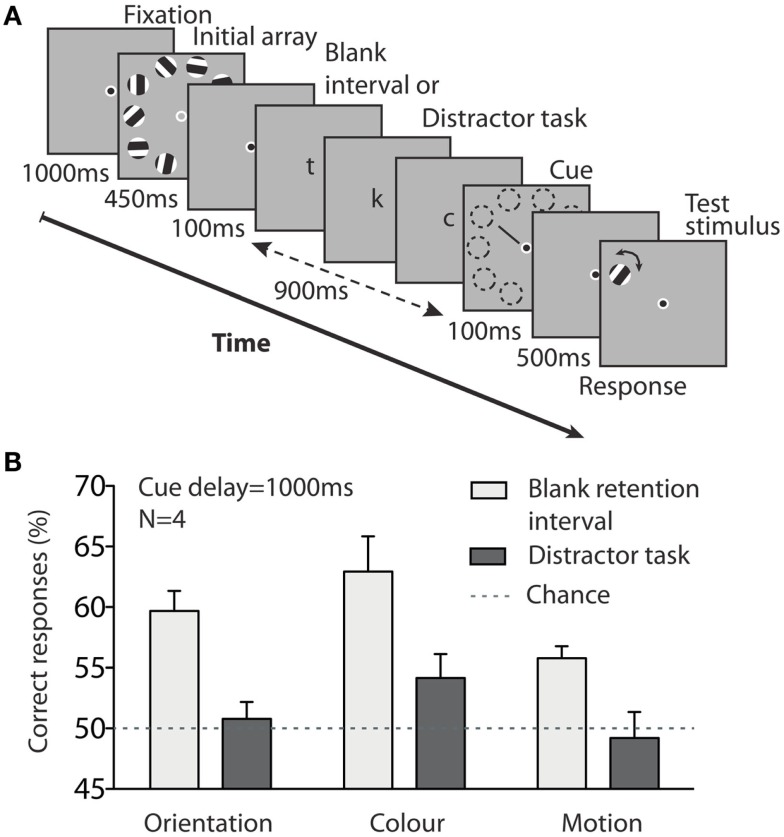
**Experiment 2 tested memory for color, form, and motion for a cue delay of 1000 ms, with or without manipulation of attention**. **(A)** On trials that contained a distractor task, participants had to detect targets in a stream of rapidly presented letters during the cue delay. A sound warned them in advance about what type of trial would occur. **(B)** Having to perform an attention-demanding task during the retention interval disrupted memory. Error bars denote standard errors of the mean, *N* = 4.

To investigate this prospect we ran a new experiment that focused on a cue delay of 1000 ms, for all three visual features. A tone before the trial indicated that a concurrent letter detection task would follow during the memory retention period. During the retention interval, attentional resources were directed to the center of the screen using an RSVP (rapid serial visual presentation) letter detection task (Figure 4A). If iconic memory supported performance at 1 s, we might expect the task to leave performance unaffected. However, if visual working memory was responsible for the performance at 1 s, we might expect task performance to drop.

Figure 4B shows the data for the three visual features. A two-way repeated-measures ANOVA (feature × distractor task) revealed a main effect of distractor task [*F*(1, 3) = 23.9, *P* = 0.016]. There was also a main effect of feature [*F*(2, 6) = 6.9, *P* = 0.028], but no interaction between feature and distractor task [*F*(2, 6) = 0.2, *P* = 0.8]. This suggests that the application of attention to the letter task degraded memory at 1000 ms by interfering with the retention process.

Here, subjects were informed about the trial type (distractor task/no distractor task) by a tone at the outset of each trial. Hence, it is possible that pre-emptive attentional allocation – participants prematurely attending centrally prior the start of the distractor task – could influence perceptual encoding of the initial array. However, participants were informed that performance on both the memory and distractor tasks was important and neither was to be prioritized. Hence, it seems unlikely that participants would prematurely attend away from the initial memory array to the degree that perceptual encoding might be degraded. If attentional resources were prematurely allocated to the central location for the upcoming letter detection task, and hence away from the initial perceptual array, it could be hypothesized that the effective contrast of the array might have been reduced as attention has been shown to alter perceived contrast. It is worth noting that in such scenarios the brightness and contrast of stimuli has been shown to have little effect on memory or in fact the inverse relationship, e.g., the lower the contrast in the stimulus the better the visual memory performance. Hence, it follows that a reduction in effective contrast in the perceptual stimuli due to premature attentional allocation might actually boost memory performance, not reduce it. Accordingly we feel that it is unlikely that a premature attentional allocation could fully account for the decline in memory accuracy we observed.

Manipulating attention (either during encoding or retention, or both) thus reduced performance at 1000 ms. Iconic memory is often thought to be an automatic high-resolution memory (Neisser, 1967), whereas visual working memory is a more effortful active process (Awh et al., 2006; Fougnie, 2008). Performance here seems dependent on attentional resources. Hence, these data suggest that retention of information regarding the 10-item array over 1000 ms might be largely due to an active process like visual working memory.

Experiment 2 suggested that visual working memory plays a role in retention of visual information at times of 1 s and above. We wanted to investigate the relationship between different visual memory stores for time periods of 1 s and less. It has been proposed that information in iconic memory is specific to the location in visual space (Phillips, 1974; McRae et al., 1987). In contrast, information stored in visual working memory might be partially location-dependent and partially independent of location (Dill and Fahle, 1998; Ester et al., 2009; Ong et al., 2009). A third experiment tested performance for the same delayed cue task, but only for the feature of orientation at cue delays of 200, 600, and 1000 ms. Importantly, here the test stimulus could appear in three different locations: same as cued (control), center (at fixation), or opposite-side to cued (diametrically opposite location in the periphery).

**Figure 5:**
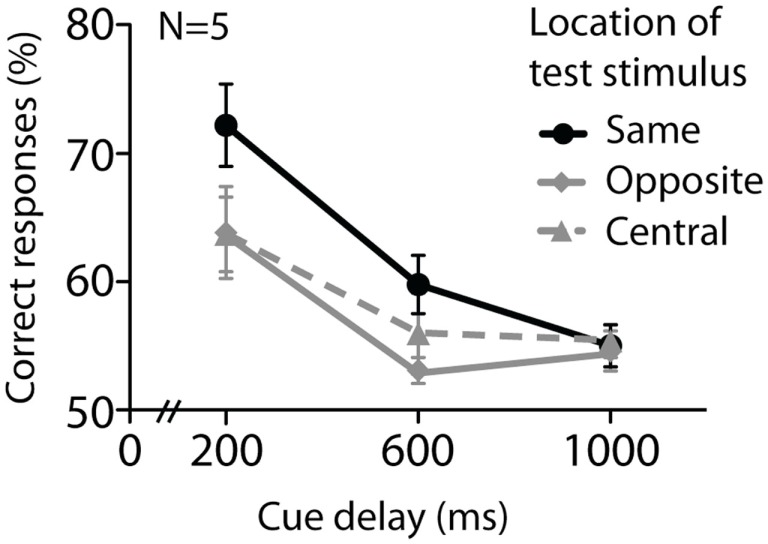
**Experiment 3 investigated the effect of test stimulus location on performance for orientation stimuli only, at 200, 600, and 1000 ms**. The test stimulus could be presented either at the same, central, or opposite location to the cued one. Performance decreased over time and was worsened if the test stimulus was presented at a central or opposite location. Error bars denote standard errors of the mean, *N* = 5.

Figure 5 shows data from Experiment 3. A two-way repeated-measures ANOVA (cue delay × test stimulus location) revealed a main effect of location of test stimulus [*F*(2, 8) = 4.7, *P* = 0.044], as well as cue delay [*F*(2, 8) = 17.3, *P* = 0.001]. However, the interaction between cue delay and test stimulus location was not significant [*F*(4, 16) = 2.4, *P* = 0.09]. Despite the difference in the two location conditions, the lack of an interaction effect makes it hard to conclusively distinguish between simple memory decay and a decrease in retinotopic representation with longer durations.

A change in the position of the test stimulus relative to the location in the original array had greater detriment to performance at shorter storage durations, although there was no significant interaction. The pattern of data is consistent with the hypothesis that information is less and less tied to a retinotopic location with longer retention times. As retinotopy is a visual characteristic of early visual cortex that is lost with accession through the visual system (Wandell et al., 2007), our data are consistent with the idea that early visual cortex might be recruited for mnemonic storage during short delays, whereas for longer storage durations subsequent areas of visual processing, more location-independent, might come into play. Future work parametrically manipulating set size might reveal further details of this relationship.

## Discussion

Our data suggest that precise information about multiple low-level visual stimuli can be retained for over 1 s. The amount of information stored in iconic memory was less for motion compared to orientation or color stimuli. Performance at 1000 ms was liable to disruption by a reallocation of attentional resources, suggesting that storage at around 1 s resembles the active process of visual working memory. Finally, memory for orientation seemed to be influenced by the retinotopic location of the test stimulus, suggesting the involvement of early retinotopic visual cortex.

Most studies of visual memory for low-level features have investigated color, orientation, and motion in separate experiments with different stimuli and subjects (Pasternak and Greenlee, 2005). In contrast, our study compared early visual memory for color, form, and motion in a single paradigm and set of experimental conditions. We found that the amount of information stored in iconic memory was significantly less for motion than for color and orientation. In contrast, long-term memory is actually boosted by the addition of moving information (Matthews et al., 2007). For intermediate delays of around 10 s memory for a single motion stimulus shows little decay (Blake et al., 1997; Pasternak and Greenlee, 2005). Why might this high-capacity memory for multiple motion stimuli show less information storage compared to color or orientation?

One proposal is that motion perception requires more visual information, in the sense of visual features or details (position changes over time), compared to color and orientation. For example motion perception has been modeled as spatiotemporal orientation, or orientation in space and time (Adelson and Bergen, 1985). Likewise, although visual search is not a direct measure of the amount of information in a visual stimulus, it is often used as an index of information load of a stimulus object (Treisman, 1986; Alvarez and Cavanagh, 2004). Indeed, the search rate, i.e., the amount of extra search-time for each additional item in visual search tasks has been shown to be greater for motion compared to color or form stimuli (Cavanagh et al., 1990; Driver et al., 1992). If motion perception does indeed require more information than other stationary visual features, then iconic memory for motion might also be contingent on greater amounts of information storage. Such a model for visual motion memory suggests that the capacity limits for memory storage of motion information would be reached with fewer overall items than other visual features like color or orientation. Such capacity limits could help explain the different decay function we found here for motion storage.

It has been proposed that after-images may largely account for iconic memory performance at particularly short cue delays (Sakitt, 1976; Sligte et al., 2008). This could potentially create discrepancies between stimuli that are not followed by after-images (e.g., motion), stimuli that produce negative after-images (color) and stimuli that are followed by informative after-images (orientation). We took this into account by counter-phasing the orientation stimuli, in order to decrease any advantage that may otherwise arise from orientation-specific after-images. Thus, it seems our results cannot be explained by the contribution of after-images to performance. In addition, the color and orientation data were not significantly different, which would be predicted if subjects were still able to utilize orientation pattern after-images to boost performance.

It has previously been shown that low-level visual features might be held in visual working memory accurately and with little decay for up to tens of seconds (Pasternak and Greenlee, 2005). Interestingly, our data show a clear decay of iconic/visual working memory over a few seconds. This finding can be explained by current accounts of the capacity limits of visual working memory. One account specifies that the precision of the representation of each object in visual working memory decreases as the number of objects to be remembered increases (Wilken and Ma, 2004; Bays and Husain, 2008; Bays et al., 2009). Because we used an array of 10 stimuli, the resolution of the sensory object representations in our study might have been lower than in other studies, which used fewer objects. For cue delays of 1 or 2 s, precision might have been just above threshold for correct discrimination on some trials and just below on other trials. This could explain the low but above chance performance for cue delays of 1 s.

Another theory specifies that a maximum of 3–4 items can be retained in visual working memory (Luck and Vogel, 1997; Cowan, 2001; Vogel et al., 2001; Zhang and Luck, 2008). The measure commonly used to assess the number of items stored assumes that items are either perfectly remembered or totally absent from memory (Pashler, 1988). Performance on a memory task can thus be equated to a discrete number of objects that might have been selected, randomly or otherwise, from the original array. If one item out of 10 is remembered on each trial with 100% precision and the other nine items are not, there is one chance out of ten that the cue will select the item remembered perfectly (correct answer), and nine chances out of 10 that it will select a non-remembered item (correct or wrong answer, equal probability). Over many independent trials, performance would tend toward *P* = (1/10 × 100 + 9/10 × 50)/100 = 55% correct responses. Using this description of memory, a performance of 60% correct responses corresponds to two items remembered, and 65% correct responses corresponds to three objects remembered. In our study, performance for 1-s cue delays could therefore be accounted for by the retention of one (motion) to three items (color).

Previous work has shown greater visual working memory performance between color sets closer in color space compared to sets farther away or more different. In other words, the less similar the colors, the worse subjects performed. Such active interference between feature representations is an additional source of memory corruption or decay. In fact, Lin and Luck (2009) note that their finding only extends to color and that other visual features, such as motion might operate differently. Hence, it remains a possibility that cortical representations of motion could actively interfere or co-corrupt other motion representations to a greater degree than color or orientation representations. Accordingly, such a scenario remains a possible hypothesis to explain our current data.

## Conclusion

Our study suggests that low-level visual features of 10 separate items can be retained for over 1 s. The amount of information stored in iconic sensory memory was less for motion than for color or orientation stimuli. Performance at 1000 ms was found to be largely an active process, which suggests the potential involvement of visual working memory.

## Conflict of Interest Statement

The authors declare that the research was conducted in the absence of any commercial or financial relationships that could be construed as a potential conflict of interest.
